# Cardiac Responses during Picture Viewing in Young Male Patients with Schizophrenia

**DOI:** 10.1155/2012/858562

**Published:** 2012-11-10

**Authors:** Roelie J. Hempel, Julian F. Thayer, Christian H. Röder, Hugo G. van Steenis, Nico J. M. van Beveren, Joke H. M. Tulen

**Affiliations:** ^1^Department of Psychiatry, Erasmus University Medical Center, ‘s Gravendijkwal 230, 3015 CE Rotterdam, The Netherlands; ^2^School of Psychology, University of Southampton, University Road, Southampton SO17 1BJ, UK; ^3^Department of Psychology, Ohio State University, Psychology Building, 1835 Neil Avenue Mall, Columbus, OH 43210, USA

## Abstract

Previous research investigating the emotion recognition ability in patients with schizophrenia has mainly focused on the recognition of facial expressions. To broaden our understanding of emotional processes in patients with schizophrenia, this study aimed to investigate whether these patients experience and process other emotionally evocative stimuli differently from healthy participants. To investigate this, we measured the cardiac and subjective responses of 33 male patients (9 with and 24 without antipsychotic medication) and 40 male control subjects to emotion-eliciting pictures. Cardiac responses were chosen as an outcome measure because previous research has indicated that these are linked with attentional and emotional processes and provide a more objective measure than self-report measures alone. The differences in cardiac responses between patients and controls were limited to medicated patients: only the medicated patients showed significantly decreased cardiac orienting responses compared with control subjects, regardless of picture contents. These results indicate that medicated patients directed less attention towards emotion-eliciting pictures than controls. Decreased attentional resources while processing emotional evocative stimuli could lead to incorrect appraisals of the environment and may have detrimental emotional and social consequences, contributing to chronic stress levels and an increased risk for cardiovascular disease.

## 1. Introduction

An extensive amount of research papers has been published on the impaired emotional functioning of patients with schizophrenia. Most of this research has focused on the impaired ability of these patients to recognize emotions from facial expressions [1], which is crucial for forming and maintaining interpersonal relationships [2, 3], and patients with schizophrenia are known to experience difficulties with social functioning [4, 5]. 

Although schizophrenic patients seem impaired in their ability to recognize and express emotional facial expressions, they do appear to experience emotions in a way similar to healthy controls. Several studies have found that patients and controls did not differ in their subjective ratings of pleasantness and arousal when presented with emotion-eliciting pictures [6–9]. In two other studies, schizophrenic patients reported that they experienced the same amount of pleasant emotions as healthy controls, but greater amounts of unpleasant emotions in response to emotion-eliciting stimuli [10, 11]. It has also been found that schizophrenic patients experience less positive emotions and more negative emotions in response to daily stressors [12] and emotion-eliciting pictures [13], compared with healthy control subjects. This apparent discrepancy between the impaired ability of schizophrenic patients to recognize and express emotional facial expressions, yet with intact experience of emotions, needs further investigation. If schizophrenic patients experience emotions in a way similar to healthy control subjects, they are expected to perceive the emotion-eliciting stimuli in a similar way as well. This could imply that the disturbances in emotion recognition in schizophrenic patients are restricted to facial stimuli, as opposed to more general emotion-eliciting stimuli.

Another aspect of emotions is the psychophysiological response that accompanies them [14]. Psychophysiological responses offer a more objective measure of emotional and cognitive processes than self-report alone. Only a few studies have investigated the cardiac responses of patients with schizophrenia during the viewing of emotion-eliciting pictures. Two of these studies did not find any differences between the patients and healthy controls with regard to their cardiac responses [7, 9]. However, both studies used the averaged heart rate response during slide viewing relative to a 1- or 2-second baseline before picture onset, without taking into account the triphasic pattern of the cardiac response (deceleration-acceleration-deceleration [14]). Yee et al. [15] did take this pattern into account and found that only prodromal patients differed significantly from healthy controls and patients with schizophrenia in their heart rate deceleration, that is, orienting response (OR), which was more pronounced in these patients. In a study previously conducted by our group [16], we found that patients with schizophrenia hardly showed any OR to emotion-eliciting pictures. Instead, we found a substantial increase in the heart rate in patients with schizophrenia that was evoked by erotic pictures, indicating an activation of the sympathetic nervous system, which could either mean a defensive reaction to these stimuli, suggesting stimulus rejection [17], or a strong appetitive reaction. Summarizing, thus far mixed results regarding cardiac responding in schizophrenia have been reported. However, none of these studies have taken medication use into account, while it has previously been found that atypical antipsychotics decrease physiological responding to emotion-eliciting stimuli [18]. 

The aim of the present study is to broaden our knowledge about the cardiac responding and underlying motivations of patients with schizophrenia while viewing emotion-eliciting pictures. Based on previous research [16], we hypothesized that the patients with schizophrenia would show diminished OR responses in general, reflecting a deficit in the intake process of stimulus information. Because of the potential importance of antipsychotic medication use on psychophysiological responses, we analysed our data separately for medicated and antipsychotic-free patients, while controlling for baseline levels of heart rate and heart rate variability, smoking, mood, and age.

## 2. Methods

### 2.1. Subjects

In this study, 33 male patients with schizophrenia (9 antipsychotic free and 24 medicated) and 40 male control subjects participated. The demographic details of the participants are shown in Table 1. The patients were recruited from the early psychosis unit at the Department of Psychiatry of the Erasmus MC, University Medical Center Rotterdam. All patients were screened by a senior clinical psychiatrist using the Comprehensive Assessment of Symptoms and History (CASH; [19]), and included in the study if they were diagnosed as suffering from schizophrenia according to the criteria of the DSM-IV [20]. All patients experienced a psychotic episode at the time of the study. All patients were inpatients and most of them were experiencing their first psychotic episode or were receiving treatment for the first time. In order to assess symptom severity, the Dutch translation of the Positive and Negative Syndrome Scale (PANSS; [21]) was used. As can be seen in Table 1, antipsychotic-free patients had higher scores for all subscales as well as the total score of the PANSS than medicated patients (positive: *t*[28] = 2.4, *P* < 0.05; negative *t*[28] = 2.6, *P* < 0.05; general psychopathology *t*[28] = 2.5, *P* < 0.05; total score *t*[28] = 3.5, *P* < 0.01). The other difference between these two groups was that the duration of illness was significantly shorter in antipsychotic-free versus medicated patients (*t*[25.96] = 3.3, *P* < 0.01). 

Antipsychotic treatment was administered according to the clinical treatment protocol of the Erasmus MC Clinic for psychotic disorders, which is congruent with both the schizophrenia treatment guidelines of the Dutch Psychiatric Association and the American Psychiatric Association. The details on medication use are shown in Table 2. 

 Controls were recruited by means of advertisements. All controls were healthy and drug free at the time of testing as assessed by means of a short phone interview and a structured questionnaire. None of the controls or their first-degree relatives had experienced past or current psychiatric illnesses. Exclusion criteria for both patients and controls were respiratory diseases, cardiovascular diseases, and the use of medication that could influence the autonomic nervous system. There were significantly more smokers in both patient groups than in the control group (*χ* ^2^ = 27.78,  *P* < 0.001).

 After the subjects were given a complete description of the study, written informed consent was obtained. The study was approved by the Medical Ethics Committee of the Erasmus MC, University Medical Center Rotterdam, and was carried out in accordance with the Declaration of Helsinki. 

### 2.2. Procedure

All experimental sessions took place between 09.00 and 11.00 hrs in the morning. The subject sat in a comfortable chair in a small, sound-attenuated dimly lit room. The experimenter was in the room with the participants at all times to ensure their safety and to make sure they understood and complied with the task instructions. Both patients and controls were asked not to use any coffee or cigarettes before and during testing on the day of the experimental session. Before the application of the electrodes participants were asked to complete a Dutch shortened version of the Profile of Mood States (POMS; [22]. Electrodes for the recordings of the heart rate were applied according to the standard laboratory procedures. After the application of the electrodes, the experimental session began with a rest period of five minutes in which subjects were asked to relax and not to speak. After this first rest period, the headphones were put on and the emotional picture task was explained. The duration of the task was approximately 25 minutes. After completion, all electrodes were removed from the subjects and they were asked to do the subjective rating task, which lasted approximately 15 minutes. All tasks were designed using E-prime (Psychology Software Tools, Inc., 2002).

### 2.3. Experimental Tasks

#### 2.3.1. Emotional Picture Task

Subjects were seated approximately 75 cm from the computer screen. All subjects were asked to relax, to breathe regularly, and not to speak during the task. The emotional picture task was a part of a larger study in which we also investigated acoustic startle responses. The subjects were told that they were about to view a series of different pictures and that loud noises were occasionally presented through the headphones. They were asked to look at the pictures the entire time they were presented on the screen, but that they did not have to respond. Forty-eight pictures were selected from the International Affective Picture System (IAPS [23]). (Positive IAPS pictures: 4220, 4290, 4608, 4660, 4670, 4680, 5260, 5470, 5621, 5910, 8030, 8170, 8490, 8501. Neutral IAPS pictures: 5120, 5260, 5510, 5530, 5535, 5711, 5731, 5740, 5900, 7000, 7002, 7004, 7006, 7009, 7010, 7020, 7025. Negative IAPS pictures: 3000, 3010, 3060, 3069, 3080, 3102, 3120, 3170, 6200, 6212, 6230, 6260, 6300, 6313, 6350, 6550.) The stimuli were chosen on the basis of their normative ratings provided with the IAPS and could be divided into six different categories, based on their contents. The positive pictures had erotic and adventure contents, the neutral pictures showed nature scenes and household objects, and the negative pictures contained pictures with mutilation and threat contents. The pictures were presented using a Dell Dimension M200a Personal Computer with a Pentium processor and a 17-inch Samsung SyncMaster monitor. 

Each picture was presented during 6 seconds, with an intertrial interval (ITI) ranging from 12 to 25 seconds. A total of 48 pictures were presented; during 12 of these pictures (4 positive, 4 neutral, and 4 negative) no startle stimulus was presented. During the remaining 36 pictures, startle stimuli were presented either 300, 800, 1300, or 3800 ms after picture onset in such a way that each of these latency conditions occurred 3 times during the viewing of positive, neutral, and negative pictures. In addition, 12 startle stimuli were randomly presented during the inter trial intervals. Thus, a total of 48 pictures and 48 startle stimuli were presented. All pictures and startle stimuli were presented completely at random so that each subject was presented with a different order of stimuli. For the purpose of this paper, we will only report the cardiac responses to the pictures that were presented without a startle stimulus. 

#### 2.3.2. Subjective Rating Task

The pictures that were presented during the startle task were again presented in the subjective rating task, using a randomized order different from that of the startle task. The participants were asked to rate how they felt during the viewing of the pictures. They responded with their dominant hand using the numbers on the keyboard. A fixation cross was presented for 3 seconds on a 17-inch computer screen, followed by a 6-second presentation of a picture. After picture offset, the subjects were asked to rate each picture using the SAM (Self-Assessment Manikin; [24]. The SAM consisted of two subsequent screens, each containing five figures. On the first screen, the figures represented the degree of pleasantness of the pictures—ranging from very unpleasant to very pleasant—on a scale from 1 to 9. On the second screen, each figure represented the degree of arousal associated with each picture—ranging from very calm to very arousing—also on a scale from 1 to 9. After their response, the fixation cross appeared again for 3 seconds, followed by the next picture. 

#### 2.3.3. Cardiac Responses

During the experiment, continuous measurements were made of the heart rate (HR). HR was recorded using a precordial lead and was sampled at 512 Hz. All data were sampled and stored on a flashcard by means of a portable digital recorder (Vitaport System; TEMEC Instruments B.V., Kerkrade, The Netherlands). Upon completion of the recording, all physiological data were imported and processed on a Personal Computer using a Vitascore software module (TEMEC Instruments BV, Kerkrade, The Netherlands). Subsequently, the interbeat intervals were calculated using R-top detection. A low pass filtered cardiac event series (LPFCES; [25]) was calculated, using a low pass filter of 0.5 Hz. This method is based on the Integral Pulse Frequency Modulation model [25]. 

The responses to the 12 pictures that were presented without a startle stimulus were used for the analyses of the cardiac responses. Data series for each individual stimulus were extracted for the pictures starting 2 seconds before stimulus onset and ending 6 seconds after stimulus onset. Cardiac responses for each stimulus were calculated by subtracting the 6 seconds after stimulus onset from the 2-second baseline period before each stimulus. This resulted in time series consisting of difference scores for each separate stimulus. 

#### 2.3.4. Baseline HR and Heart Rate Variability (HRV)

Mean HR was calculated over a 3-minute period of rest. Additionally, this HR time series was subjected to a discrete Fourier transform (CARSPAN program, Groningen, The Netherlands [26, 27]), to yield power spectra of the rhythmic oscillations over a frequency range of 0.02–0.50 Hz, with a resolution of 0.01 Hz. The power was calculated for the high frequency band (HF-HRV: 0.15–0.5 Hz). Because of the skewed distribution, a natural log transformation was applied to HF-HRV. 

### 2.4. Statistical Analyses

All analyses were conducted using SPSS version 13.0 and MlWin 2.10 [28]. All alpha's were set at 0.05 unless otherwise specified. All self-report responses were used as a manipulation check and to make sure participants had complied with the task instructions.

#### 2.4.1. Profile of Mood States (POMS)

To investigate the subjective mood state of the participants at the time of testing, we analysed their POMS scores using nonparametric tests. To assess whether there were any differences between the three groups (medicated, antipsychotic free, and controls), the Kruskal-Wallis test was used. When significant differences were found, we proceeded to investigate which groups differed from each other using the Mann-Whitney tests and to correct for multiple testing, the alpha was reduced to 0.016. The maximal scores for each of the scales were as follows: depression 32, fatigue 24, tension 24, anger 28, and vigour 20. The Total Mood Disturbance (TMD) score was calculated by subtracting the vigour score from the sum of all the other scores (i.e., depression + fatigue + tension + anger − vigour = TMD), so that a higher positive score represented a more negative mood state. 

#### 2.4.2. Cardiac Responses

**Baseline**

The mean HR level and the ln(HF-HRV) of the three groups during 3 minutes of resting baseline before the onset of the tasks were compared using an analysis of variance (ANOVA) with Group (medicated, antipsychotic-free, and controls) as the between subjects factor.

**Cardiac Response to Picture Stimuli**

For the analysis of cardiac responses to the emotional stimuli, we developed an hierarchical linear model (HLM) using MlWin software [28], with Subject, Time, and Picture Type entered as repeated variables. The fixed factors in the HLM were Group (medication free, medicated, and control), Time (0–6 seconds, in half second bins) with its linear and quadratic polynomials, Picture Type (negative, neutral, and positive), Group × Time, Group × Picture Type, Time × Picture Type, and Group × Time × Picture Type. The following covariates were entered smoking (number of cigarettes per day), age, illness duration (months), medication duration (weeks), POMS TMD score, baseline ln(HF-HRV), and baseline HR. We also entered the following interaction effects: ln(HF-HRV) × Time and baseline HR × Time; these were included to examine whether they were related to a different cardiac responding to startle stimuli, since we previously found that schizophrenic patients generally experience decreased levels of HF-HRV [29], which could explain overall decreased cardiac responsivity to stimuli. All factors and covariates were entered in the model and the best fit for the model was sought using the log-likelihood method. Significant interaction effects were followed up by separate linear models.

#### 2.4.3. SAM Ratings

Since the Kolmogorov-Smirnov tests indicated that the distributions of the SAM rating of pleasure and arousal were normally distributed within the groups (*P* values ranged from 0.13 to 1.00), we performed two repeated measures ANOVA's with Pleasure or Arousal rating as the dependent variable, Group (medicated, antipsychotic-free and controls) as the between subjects factor, and Picture Type as the within subjects factor. 

## 3. Results

### 3.1. Profile of Mood States (POMS)

The POMS scores of the three groups are reported in Table 3. Because the Kruskal-Wallis test showed that there were significant differences between the three groups in mood scores (depression *χ*_2_^2^ = 28.0, *P* < 0.001; fatigue *χ*_2_^2^ = 9.7, *P* < 0.01; tension *χ*_2_^2^ = 22.0, *P* < 0.001; anger *χ*_2_^2^ = 16.9, *P* < 0.001; vigor *χ*_2_^2^ = 6.8, *P* < 0.05; TMD score *χ*_2_^2^ = 23.2, *P* < 0.001), we used the Mann-Whitney tests to further explore these differences. The medicated and antipsychotic-free patients did not differ significantly in their mood scores for any of the subscales (all *P*'s were 0.10 or higher). However, compared to control subjects, the antipsychotic-free patients showed significantly decreased mood on all subscales and the TMD score (all *P*'s < 0.01), and the medicated patients had significantly decreased mood scores for depression, tension, anger, and the TMD score (all *P*'s < 0.001). 

### 3.2. Heart Rate and HF-HRV at Baseline

Data was lost for one medicated patient due to excessive movements during baseline. We found a significant Group effect when we investigated whether the resting HR of patients who were not under current antipsychotic medication (*n* = 9) and medicated patients (*n* = 23) differed from the resting HR of the control group (*n* = 40), *F*[2,69] = 8.26, *P* = 0.001, partial *η* ^2^ = 0.19. The mean HR of medicated patients was 83 ± 16 bpm, of antipsychotic-free patients 75 ± 13 bpm, and of controls 69 ± 11 bpm. The Bonferroni post hoc tests indicated that only the mean HR of the medicated patients was significantly higher than that of the control subjects (mean difference = 14.05, sd = 3.46, *P* < 0.001).

We also found a significant Group effect for the natural logarithm of HF-HRV, *F*[2,69] = 6.07, *P* < 0.01, partial *η* ^2^ = 0.15. The Bonferroni post-hoc test (mean difference = 1.08, sd = 0.31, *P* < 0.01) showed that medicated patients had a significantly lower HF-HRV (6.21 ± 1.59) than the control subjects (7.29 ± 0.92), while the antipsychotic-free patients did not differ significantly from either groups (7.18 ± 1.17). 

### 3.3. Cardiac Response to Picture Stimuli

The following factors did not contribute significantly to the model and were therefore removed: the three-way interaction between Group × Time × Picture Type, Smoking, and the interaction between baseline HR × Time. 

**Figure 1:**
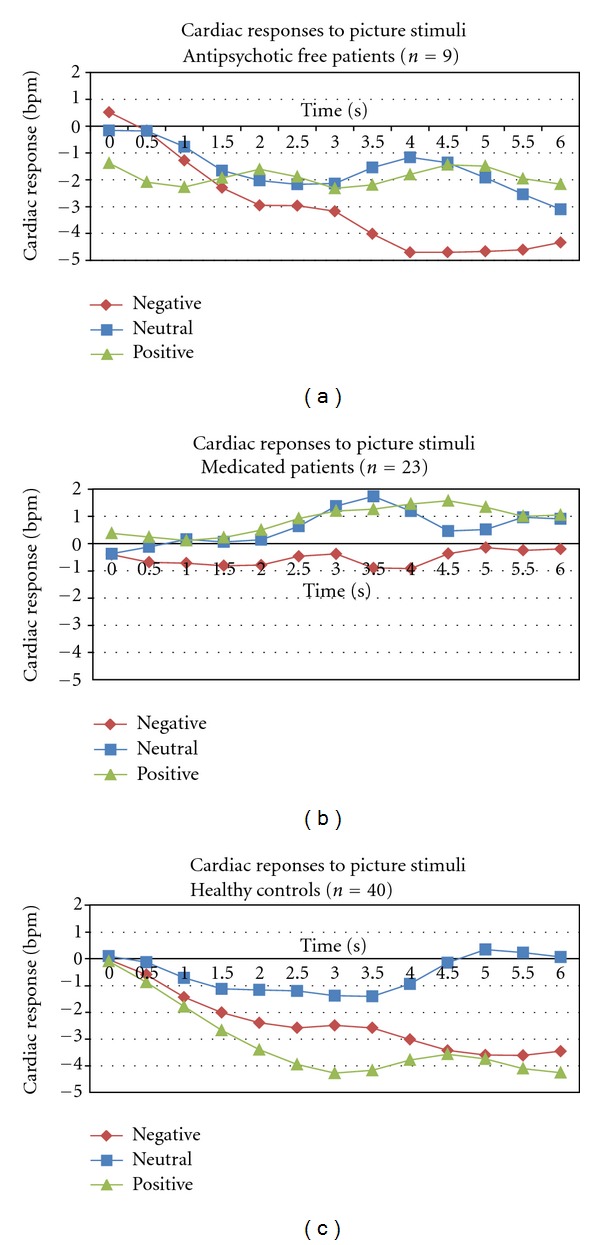
Time series of mean cardiac responses to negative, neutral, and positive picture stimuli in (a) antipsychotic-free patients, (b) medicated patients, and (c) healthy control participants during the 6 seconds of picture presentation.

 All beta-coefficients for this model are reported in Table 4, and the cardiac responses of each of the three groups are shown in Figures 1(a)–1(c). The significant main effect for Group (joint *χ*_(1)_^2^ = 94.41, *P* < 0.01) indicated that all three groups differed from each other in terms of overall HR level: with all other variables being constant, the medicated patients showed the least deceleration, followed by the controls, while the antipsychotic-free patients showed the most deceleration overall. As expected, the main effect for Picture Type (joint *χ*_(1)_^2^ = 11.04, *P* < 0.01) indicated that the HR decelerations were significantly larger for negative and positive pictures compared to neutral pictures. The significant main effect of Time linear showed that HR decreased over time. 

 The significant interaction effect of Group × Picture Type (joint *χ*_(1)_^2^ = 23.50, *P* < 0.01) indicated that the HR responses to the various picture types of the patient groups were less differentiated than the HR responses of the control group. For example, the difference in HR deceleration between negative and neutral pictures was smaller in the medicated group compared to the control group, while the difference in HR response between positive and neutral pictures was smaller in the antipsychotic-free group compared to the control group. Also, the medicated patients showed more HR deceleration to positive versus negative pictures, whereas both the antipsychotic-free patients and the controls did not. The significant interaction effect of Group × Time^1^ (joint *χ*_(1)_^2^ = 10.80, *P* < 0.01) indicated that medicated patients showed significantly less HR deceleration over time than antipsychotic-free patients and healthy controls. The Picture Type by Time^1^ (joint *χ*_(1)_^2^ = 24.98, *P* < 0.01) interaction effect confirmed that HR decelerates more over time for negative and positive pictures than neutral pictures. 

 With regard to the covariates, the significant effect of Age indicated that the HR increased with age, whereas the significant effects of duration of medication use and illness duration indicated more deceleration with longer antipsychotic medication use and/or longer illness duration. We also found that Baseline HR was negatively related to cardiac response: a higher HR at baseline is related to more HR deceleration during picture viewing, and ln(HF-HRV) significantly interacted with Time^1^, indicating that increases in cardiac vagal activity during rest were related to an increased cardiac response. Finally, increased negative mood was related to a cardiac increase, as shown by the significant effects of the POMS TMD score. 

### 3.4. Follow-Up Analyses per Group

Because of the significant two-way interaction effects with Group and because the figures suggested a decreased orienting response in the patient groups to positive and negative compared to neutral pictures, we further investigated this with follow-up HLM models for each group, only including the cardiac responses during the first 3 seconds of picture viewing. 

 Within the antipsychotic free patient group, the Time × Picture Type interaction effect was significant (joint *χ*_(1)_^2^ = 9.31, *P* < 0.01), indicating that the heart rate decreased more over time for negative than for positive pictures, that is, the antipsychotic-free patients showed a larger orienting response to negative versus positive pictures. Within the medicated patient group, the main effect for Picture Type was significant (joint *χ*_(1)_^2^ = 5.33, *P* < 0.01), indicating that the heart rate of the medicated patients was lower during the first 3 seconds for positive compared to neutral pictures. Within the control group, the main effects for Time (*Z* = −3.92, *P* < 0.01) and Picture Type (joint *χ*_(1)_^2^ = 22.47, *P* < 0.01). As can be seen in Figure 1(c), the HR of the controls decreased more for positive and negative than for neutral pictures. The interaction effect of Time × Picture Type (joint *χ*_(1)_^2^ = 6.30, *P* < 0.01) was also significant, although pairwise comparisons were not. 

### 3.5. Subjective Ratings

**Figure 2:**
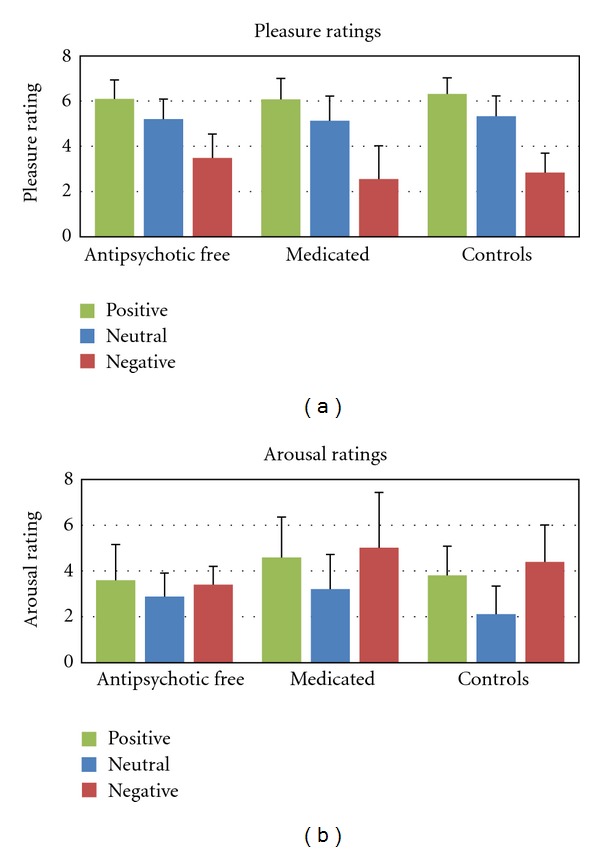
(a) Pleasure and (b) Arousal ratings of antipsychotic-free patients, medicated patients, and healthy control subjects for positive, neutral, and negative pictures.

In Figures 2(a) and 2(b), the mean SAM ratings for pleasure and arousal are presented. For the pleasure ratings, we found a significant main effect for Picture Type (*F*[1.7; 121.6] = 178.88, *P* < 0.001, partial *η* ^2^ = 0.72). The positive pictures were rated as most pleasant, followed by the neutral pictures, and the negative pictures were rated as least pleasant (positive versus neutral: *F*[1,71] = 47.32, *P* < 0.001, partial *η* ^2^ = 0.40; positive versus negative: *F*[1,71] = 296.50, *P* < 0.001, partial *η* ^2^ = 0.81; neutral versus negative: *F*[1,71] = 136.00, *P* < 0.001, partial *η* ^3^ = 0.66). No significant main or interaction effects for Group were found.

 For the arousal ratings a significant main effect for Picture Type was found (*F*[1.6; 108.5] = 28.68, *P* < 0.001, partial *η* ^2^ = 0.29). The negative and positive pictures were rated as more arousing than the neutral pictures, whereas no difference in arousal ratings was found between positive and negative pictures (negative versus positive: *F*[1,70] = 1.23, *P* = 0.27; negative versus neutral: *F*[1,70] = 39.38, *P* < 0.001, partial *η* ^2^ = 0.36; positive versus neutral: *F*[1,71] = 74.17, *P* < 0.001, partial *η* ^2^ = 0.51). The main effect for Group was also significant (*F*[2,70] = 3.61, *P* < 0.05, partial *η* ^2^ = 0.09). The Games-Howell test showed that the arousal ratings of the medicated patients group tended to be higher than those of the antipsychotic-free and control groups (medicated versus antipsychotic-free: mean difference = 0.98, sd = 0.44, *P* = 0.09; medicated versus control: mean difference = 0.83, sd = 0.37, *P* = 0.07). The interaction effect between Picture Type and Group almost reached significance (*F*[3.1; 108.5] = 2.49, *P* = 0.06, partial *η* ^2^ = 0.07). The antipsychotic-free patients tended to show less differentiation in arousal ratings between the negative and neutral, and positive and neutral pictures compared with both the medicated and control groups (negative versus neutral: *F*[2,70] = 3.87, *P* < 0.05, partial *η* ^2^ = 0.10; positive versus neutral: *F*[2,70] = 3.46, *P* < 0.05, partial *η* ^2^ = 0.09).

## 4. Discussion

The aim of this study was to investigate whether male patients with schizophrenia differed from healthy control subjects in their psychophysiological responses to emotion-eliciting pictures. A distinction was made between patients who were taking antipsychotic medication and patients who were not. 

 While the healthy control subjects and antipsychotic-free patients showed the expected triphasic pattern (i.e., decrease-increase-decrease) for cardiac responses to positive and negative pictures—albeit somewhat less pronounced for positive pictures in antipsychotic-free patients—the medicated patients did not show an initial decrease for any of the picture types, suggesting a lack of orientation towards the pictures. The orientation response (OR), the initial deceleration of the heart rate, is considered to be the basic cardiac response to perceptual stimuli and tends to be greatest for unpleasant pictures. The cardiac OR is thought to reflect the intake of stimulus information [30–32]. Our results suggest that in medicated patients with schizophrenia this OR is lacking a finding that is consistent with the existing literature on attentional deficits in these patients [33–35]. However, we did not find this lack of OR in antipsychotic-free patients. It seems paradoxical that the antipsychotic-free patients showed overall decreased mood scores and more severe symptom scores yet physiologically they seemed to respond more similarly to healthy control subjects than the medicated patients. A number of factors could account for this finding. 

First, a difference in sympathovagal balance in the patient group could account for the difference in ORs. Previous studies have consistently found a decrease in cardiovagal control in patients with schizophrenia, regardless of medication status [36–40]. Indeed, we found that the power of high frequency HRV was lower in medicated patients compared with healthy control subjects, but not antipsychotic-free patients. The initial HR deceleration related to orienting is generally considered to be under vagal control [41–43]. The autonomic nervous system (ANS) is considered the link between the central nervous system and the body, and so the ANS mediates the relationship between centrally occurring events such as emotions and bodily changes such as heart rate variability [44]. Thus, generally reduced cardio-vagal control in patients may have caused the reduced OR rather than this being a result of decreased emotional experience or attention in response to emotion-eliciting pictures. However, after controlling for reduced cardio-vagal control by including HRV as a covariate in our analyses the differences between the medicated and control groups were still significant, suggesting that a difference in central processing was at least partially responsible for the different cardiac responses between groups. 

Second, the sample size for the antipsychotic-free patients was very small, so the lack of a significant difference between antipsychotic-free patients and healthy control subjects could be due to a lack of statistical power. Although we cannot rule out this possibility, Figures 1(a)–1(c) suggest that antipsychotic-free patients had heart rate decelerations during picture viewing comparable to controls, and a previous study [15] showed an almost identical pattern of cardiac responses to emotion-eliciting pictures in a group of prodromal patients (*n* = 12, of which 8 are antipsychotic free). Third, the use of antipsychotic medication may have caused side effects such as sedation and/or drowsiness. However, the medicated patients did not differ significantly in their subjective fatigue and vigour scores of the POMS when compared to healthy controls. Thus, a number of factors could have caused this difference between medicated and antipsychotic-free patients, but with our current data we are unable to answer this question satisfactorily. We therefore propose that future research should focus more on the difference in emotional responding between medicated and antipsychotic-free patients with schizophrenia to disentangle medication side effects from emotional processing deficits. 

 Medicated and antipsychotic-free patients differed significantly with regard to the subjective arousal ratings of the pictures. Medicated patients tended to show overall higher arousal ratings than antipsychotic-free patients and controls, a finding that is in line with the previous study of Schlenker et al. [7]. We also found that antipsychotic-free patients showed less differentiation in their arousal scores of neutral versus negative and positive pictures than medicated patients and healthy controls. Whether this finding is caused by increased arousal ratings to neutral pictures or decreased arousal ratings to positive and negative pictures is not clear at the moment. All three groups rated the pictures similarly in terms of pleasure, with positive pictures receiving the highest and unpleasant pictures receiving the lowest pleasure scores. 

There were some limitations to our study. For example, we only included male participants in this study, which reduces the generalization of our results. Also, we did not differentiate between paranoid and nonparanoid patients in our analyses. Rather, we chose to investigate a more heterogeneous patient sample to be able to generalize our results to a patient population that suffered from recent-onset schizophrenia, regardless of specific symptoms, that is, patients with predominantly positive, negative, or disorganized symptoms were all included in this study. 

As it is a common practice in studies investigating psychophysiological responses to emotion-eliciting pictures, we sampled and analysed the cardiac responses only for the time period in which the picture was presented, that is, for 6 seconds. However, because we did not analyse the cardiac responses after picture offset it is possible we have missed potential differences between groups. Previous studies measuring different types of responses (i.e., behavioural, startle, and fMRI) have found evidence to suggest that although patients' responses do not differ from healthy control subjects while an emotionally evocative stimulus is present, they do appear to have difficulties in maintaining the elicited emotional response once the stimulus disappears [45–47], thereby reducing their ability to use emotions to help them select the most appropriate behavioural response. Because we asked our participants to give their subjective ratings immediately after picture offset we were unable to analyse the responses after picture offset. Future studies should allow for the investigation of maintained emotional and physiological responses to increase our understanding of the emotional and motivational challenges patients with schizophrenia experience. 

One potential weakness of this study is that, like most studies investigating cardiac responses to picture stimuli, we did not control for respiration. Asking participants to pace their breathing would have been too demanding and would have confounded our results. In addition, opinions with regard to the usefulness and validity of controlling respiration while measuring cardiac vagal control and/or cardiac responses are divided [48–50]. We tried to control for respiratory sinus arrhythmia by entering HRV as a covariate but a more sophisticated within-subject analysis beyond the scope of this study would be required to control for respiratory variations in the cardiac time series [48]. 

## 5. Conclusions and Implications

Our results indicate that the main difference between patients with schizophrenia and healthy control subjects lies in the reduced amount of attentional resources available in medicated patients while viewing emotion-eliciting pictures. No interaction effects between emotional picture contents and groups were found, indicating that this is a general effect and not caused by any specific picture content. These results are in line with previous studies in which no differences in cardiac responding between patients and controls were found [7, 9]. 

We replicated our previous findings regarding the lack of orienting responses in patients with schizophrenia [16]. The decreased OR we found in our patient sample is in line with findings that patients with schizophrenia gather little information before responding to certain tasks, a response style termed “jumping to conclusions” [51, 52]. Abnormalities in the identification of emotionally salient information may lead to misinterpretations of the intentions of others, which may increase or exacerbate paranoid thoughts and result in dysfunctions in social behaviour [53]. 

Medicated patients also showed emotionally dampened responses: that is, not only did they show a reduced or absent OR but they also had less differentiation in their responses to the emotion-eliciting pictures. McCubbin et al. [54] propose that emotional dampening could increase psychological distress via inappropriate social interactions with family members, friends, and coworkers. In addition, emotional dampening has been found to be related to increased risk for cardiovascular disease, most notably hypertension [54]. 

Summarizing the combination of decreased stimulus intake, jumping to conclusions and emotional dampening can all contribute to chronic stress levels in patients with schizophrenia, resulting in difficulties in social and emotional functioning and an increased risk for cardiovascular disease. 

## Figures and Tables

**Table 1:** Demographic characteristics of the patient and control groups.

	Medicated patients (*n* = 24)	Antipsychotic-free patients (*n* = 9)	Controls (*n* = 40)
Age (mean ± Sd)	22 ± 4	21 ± 6	23 ± 4
Smokers (Yes/No)^a^	20/4	7/2	8/32
Duration of illness (months ± Sd)^b^	16 ± 19	3 ± 3	
Duration of current medication use (weeks ± Sd)	4 ± 4		
PANSS^c ^(mean ± Sd)			
Positive symptoms	17 ± 6	23 ± 6	
Negative symptoms	15 ± 5	21 ± 5	
General psychopathology	33 ± 9	43 ± 12	

Total	65 ± 16	87 ± 14	

PANSS: positive and negative syndrome scale; Sd: standard deviation.

^a^There were significantly more smokers in both patient groups than in the control group (*χ* ^²^ = 27.78, *P* < 0.001).

^b ^The duration of illness was significantly shorter in antipsychotic-free versus medicated patients (*t*[25.96] = 3.3, *P* < 0.01).

^c^Antipsychotic-free patients had higher PANSS scores for all subscales than medicated patients (positive: *t*[28] = 2.4, *P* < 0.05; negative *t*[28] = 2.6, *P* < 0.05; general psychopathology *t*[28] = 2.5, *P* < 0.05; total score *t*[28] = 3.5, *P* < 0.01).

**Table 2:** Mean dosages and duration of current antipsychotic treatment of the patient group.

Antipsychotic	*n*	Mean dosage (mg) ± Sd	Comedication
No Antipsychotic	9		Lorazepam (*n* = 3)
Olanzapine	8	16.3 ± 6.9	Lorazepam (*n* = 2)
			Oxazepam (*n* = 1)
Risperidone	6	3.0 ± 0.8	Lorazepam (*n* = 1)
			Oxazepam (*n* = 1)
Haloperidol	4	2.8 ± 1.5	Lorazepam (*n* = 2)
Clozapine	4	300.0 ± 81.6	Lorazepam (*n* = 3)
Quetiapine	2	450.0 ± 212.13	—

Sd: standard deviation.

**Table 3:** Profile of mood states scores per group.

	Medicated patients (*n* = 24)	Antipsychotic-free patients (*n* = 9)	Controls (*n* = 40)
POMS mean ± Sd (range)			
Depression	7 ± 7 (0–22)*	8 ± 9 (0–28)*	1 ± 2 (0–6)
Fatigue	6 ± 6 (0–18)	8 ± 5 (1–15)*	3 ± 3 (0–11)
Tension	6 ± 5 (0–16)*	6 ± 4 (0–12)*	2 ± 2 (0–9)
Anger	5 ± 5 (0–17)*	7 ± 5 (0–16)*	1 ± 2 (0–10)
Vigour	11 ± 5 (2–18)	7 ± 5 (0–13)*	12 ± 3 (5-18)
TMD	12 ± 20 (−15–60)*	21 ± 22 (2–68)*	−5 ± 7 (−14–14)

Sd: standard deviation; TMD: total mood disturbance; *significant difference compared to controls, *P* < 0.01.

**Table 4:** Beta coefficients of the HLM for cardiac responses to emotion-eliciting pictures in medication free patients, medicated patients, and healthy control subjects.

Factor	Comparison between GroAFPs	Comparison between picture types	Beta coefficient	Standard error	*Z*-score*
Constant			−0.13	1.11	−0.12
	AFP versus C		−0.88	0.43	**−2.04**
Group	MP versus C		1.60	0.36	**4.51**
	MP versus AFP		2.51	0.45	**5.57**
		Neg. versus Neu.	−2.12	0.24	−**8.76**
Picture type		Pos. versus Neu.	−1.79	0.24	−**7.39**
		Pos. versus Neg.	0.33	0.24	1.37
Time^1^			−5.63	1.72	−**3.27**
Time^2^			−0.03	1.72	−0.02
	AFP versus C	Neg. versus Neu.	0.59	0.56	1.05
	MP versus C	Neg. versus Neu.	1.54	0.40	**3.86**
	MP versus AFP	Neg. versus Neu.	0.92	0.60	1.54
	AFP versus C	Pos. versus Neu.	1.65	0.56	**2.93**
Group × Picture Type	MP versus C	Pos. versus Neu.	0.69	0.40	1.72
	MP versus AFP	Pos. versus Neu.	−1.02	0.60	−1.71
	AFP versus C	Pos. versus Neg.	1.06	0.56	1.89
	MP versus C	Pos. versus Neg.	−0.86	0.40	−**2.15**
	MP versus AFP	Pos. versus Neg.	−1.92	0.60	−**3.20**
	AFP versus C		−0.60	0.83	−0.72
Group × Time^1^	MP versus C		1.85	0.64	**2.92**
	MP versus AFP		2.46	0.91	**2.70**
	AFP versus C		−0.69	0.83	−0.83
Group × Time^2^	MP versus C		−1.00	0.64	−1.57
	MP versus AFP		−0.30	0.91	−0.33
		Neg. versus Neu.	−3.15	0.65	−**4.85**
Picture Type × Time		Pos. versus Neu.	−2.26	0.65	−**3.48**
		Pos. versus Neg.	0.90	0.65	1.38
		Neg. versus Neu.	0.26	0.65	0.40
Picture Type × Time		Pos. versus Neu.	0.37	0.65	0.56
		Pos. versus Neg.	0.11	0.65	0.17
Age			0.10	0.02	**4.95**
Medication duration			−0.17	0.03	−**5.09**
Illness duration			−0.02	0.01	−**2.88**
HR baseline			−0.03	0.01	−**3.71**
Ln(HRV HF)			−0.09	0.08	−1.09
Ln(HRV_HF) × Time^1^			0.82	0.23	**3.62**
Ln(HRV_HF) × Time^2^			0.18	0.23	0.79
POMS TMD score			0.01	0.01	**2.33**

*Numbers expressed in bold indicate significant effects at the 0.05 level.

HR: heart rate; Ln(HF-HRV): natural logarithm of high frequency heart rate variability; POMS: profile of mood states; TMD: total mood disturbance;

AFP: antipsychotic-free patients; C: control; MP: medicated patients; Neg: negative pictures; Neu: neutral pictures; Pos: positive pictures.
